# Characterizing Ultimatum Game responders: a scoping review of factors that influence decision-making through an evolutionary lens

**DOI:** 10.3389/fpsyg.2026.1713778

**Published:** 2026-02-09

**Authors:** Adhiraj Chowdhury, Madhavi Rangaswamy, Akash Kolte

**Affiliations:** School of Psychological Sciences, Christ University, Bengaluru, Karnataka, India

**Keywords:** emotions, fairness, inequity aversion, reciprocity, responder behavior, ultimatum game

## Abstract

The Ultimatum Game is a widely used tool for studying conflict resolution within a bargaining framework. This scoping review aims to comprehensively examine the various internal and external factors influencing the responder’s behavior in this game and compile the *status quo* of the knowledge space. 31 pertinent research articles were identified from databases like Google Scholar, PubMed and JStor, using the following keywords “ultimatum game,” “responder behavior,” “emotions and the ultimatum game,” “fairness in the ultimatum game,” “social norms and the ultimatum game,” “punishment game,” “impunity game,” “outside options in the ultimatum game.” An analysis of the same yielded two broad domains of influencing factors: internal and external. Internal factors encompassed emotions, personality traits, and cognitive capabilities, showcasing their significant influence on decision-making. External factors, including ownership, social norms, power dynamics, outside options, gender, and attraction, revealed how the context of the game shaped responder choices. This review investigates how internal and external factors influence bargaining behavior within the Ultimatum Game, distinguishing between typical and atypical responder behavior. Invoking Kahneman’s dual system theory offer insights into the evolutionary roots and modern cognitive processes guiding decision-making. The interplay between these systems reveals nuanced responses to fairness, reciprocity, and self-interest, challenging traditional economic models. While acknowledging the oversimplification of brain dynamics in these studies and also the need for cultural integration, the current review compiles a framework that advances our understanding of human behavior across disciplines, particularly for economics, psychology, and evolutionary biology. Refining this model promises deeper insights into decision-making processes amidst societal complexities.

## 1 Introduction

Decision-making, pivotal in human interaction, is a complex cognitive process influenced by various factors, such as social, emotional, and cognitive considerations (Cacioppo and Gardner, 1999; Prezenski et al., 2017). From everyday choices to global negotiations, decisions shape individual lives and society (Camerer, 2003). Bargaining, integral to decision-making, illustrates power dynamics, fairness, and strategic negotiation (Nash, 1950). The Ultimatum Game (UG) is a key paradigm in studying bargaining behavior, and used widely in experimental economics and social psychology (Güth et al., 1982).

The game involves a proposer and a responder. The proposer divides a given endowment between themself and the responder. If the responder accepts the proposed division, the sum is divided accordingly. Otherwise, both parties receive no compensation. This paradigm reveals aspects such as fairness and justice in decision-making (Van Dijk and Vermunt, 2000), exploration of human behavior complexities that include social preferences and trust (Achtziger et al., 2016; Büchner et al., 2004). Cultural differences are known to affect justice expectations (Farh et al., 1997), and personal characteristics can influence risk tolerance (Reddy and Mahapatra, 2017).

The Ultimatum Game yields diverse responses across players. Proposer behavior significantly impacts outcomes (Oosterbeek et al., 2004), while studying responder behavior quantifies decision-making (Bohnet and Zeckhauser, 2004). Understanding these factors sheds light on decision-making processes. A review by Debove et al. (2016) delve into evolving fairness models in the Ultimatum Game. In simulated bargaining, various factors influence outcomes, including social preferences (Fehr and Schmidt, 1999; Oosterbeek et al., 2004), cognitive and emotional processes (Feng et al., 2015; van ’t Wout et al., 2006), and cultural influences (Henrich et al., 2001). Investigation of these factors can help determine bargaining dynamics.

The responder’s pivotal role in any bargaining scenario is underscored by their authority to make a binary choice: to accept or reject. This binary decision encapsulates a multitude of underlying factors that intricately shape the responder’s decision-making process. This paper aims to synthesize the extensive body of literature addressing these factors and formulate a comprehensive framework that elucidates why responders engage in bargaining behaviors as they do. By analyzing these influential factors, we endeavor to offer insights into individual decision-making in daily life and into broader realms such as economic decision-making, corporate negotiations, and deal brokering (Bolton et al., 1998; Güth et al., 1982).

A focused examination of responder behavior within the context of the Ultimatum Game serves to illuminate dynamics inherent to conflict resolution (Fehr and Schmidt, 2006). The binary choice between accept and reject reveal a complex interplay of fairness perceptions, altruistic tendencies, and adherence to social norms, all of which wield considerable influence over decision-making strategies and conflict resolution methodologies (Camerer, 2003; Henrich et al., 2001). This paradigm closely mirrors real-world scenarios wherein individuals attempt to balance between personal gain and societal expectations (Gächter and Herrmann, 2009), thus shaping the trajectory of future bargaining endeavors (Fehr and Schmidt, 2006) and enriching our understanding of conflict resolution dynamics (Ostrom, 2000).

Kahneman’s dual cognitive system distinguishes between fast, automatic System 1 and slow, deliberative System 2 decision-making. The dual system model aligns well with the evolutionary framework of the “old brain” (medial frontal pole) versus “new brain” (lateral frontal pole), where the medial frontal regions underlie intuitive responses and lateral frontal regions are responsible for complex reasoning (Bakkour et al., 2019; Kahneman, 2011). In the Ultimatum Game, rejection of unfair offers is often linked to System 1’s automatic fairness preference, while acceptance aligns with self-interest driven by System 2 (Bear and Rand, 2016). Additionally, studies indicate a blend of deliberative and intuitive processes among responders, with the two cognitive systems diverging in the case of an unfair offer [as explained above by Bear and Rand (2016)] and converging toward acceptance for a fair offer (Wei et al., 2022). Hence, an evolutionary perspective enhances our understanding of responder behavior, revealing the nuanced interplay between cognitive systems in bargaining contexts.

In his seminal exploration of the Ultimatum Game, Güth and others identified a prevailing tendency among responders to accept fair offers while rejecting unfair ones, establishing a paradigm of typical behavior (Güth et al., 1982). However, subsequent research has unveiled instances of deviation from this norm, wherein participants exhibit unexpected responses by rejecting fair offers or accepting unfair ones. This departure from typicality prompted an investigation into the underlying factors influencing such behavior, particularly regarding perceptions of fairness and power dynamics. Therefore, this review endeavors to examine the existing literature to elucidate the multifaceted factors contributing to deviations from typical responses in the Ultimatum Game. The overarching objectives of this endeavor are twofold: firstly, to comprehensively understand the diverse array of factors at play within the Ultimatum Game, and secondly, to discern potential categorizations of responders based on these influential factors. By enhancing our understanding of the complexities inherent in decision-making processes, this paper aims to provide insights that contribute to a deeper understanding of bargaining dynamics and decision-making phenomena within the Ultimatum Game paradigm.

## 2 Methods

The researchers searched the following databases—Google Scholar, PubMed, JStor and Science Direct for research articles on the various factors affecting the Ultimatum Game. After an initial discussion, keywords like “emotions,” “culture,” and “reputation” and key phrases like “ultimatum game,” “responder behavior,” and “influences on the ultimatum game” were used. Upon an in-depth search of these databases 31 relevant articles were identified for analysis for the review. All searches were limited by language (English) and type of study (only empirical studies). The articles were then read thoroughly and labeled according to the influence factor being studied. All these labels were then classified into two groups based on each item being either an internal or an external factor. Any factor theme with < 2 studies matching it was not considered for the review.

## 3 Results

### 3.1 Internal factors

A substantial body of literature has been dedicated to investigating the influence of various factors on decision-making processes within the context of the Ultimatum Game. Some of these factors can be grouped into influences that emerge intrinsically which this paper will label “internal factors.”

The participants’ affective state emerged as a significant factor as Riepl et al. (2016) observed that individuals in a negative affective state were more prone to rejecting unfair offers than those in a positive affective state who exhibited a higher likelihood of accepting such offers. Collectively, these investigations underscore the influence of internal factors encompassing personality traits, affective states and social value orientations on decision-making within the Ultimatum Game. This paper has comprehensively reviewed relevant literature categorizing it into three primary domains: emotions and affective states, personality traits, and cognitive capabilities. Table 1 provides an overview of all the factors reviewed.

**TABLE 1:** Internal factors impacting responder behavior.

Author	Factor name	Behavioral drive	Response to offer	Type of behavior
Riepl et al. (2016)	Emotion state/mood	Positive mood	Accept unfair	Generous
Takagishi et al. (2016)	Fear	Risk averse approach	Reject unfair	Typical
Espín et al. (2015)	Spite	Harming others	Reject unfair	Typical
	Impatience	Minimize others’ gain	Reject unfair	Typical
Aina et al. (2020)	Anger	Response to unfair offer	Reject unfair	Typical
Gilam et al. (2019)	Anger	Response to unfair offer	Reject unfair	Typical
Iranzo et al. (2012)	Empathy	Increased acceptance threshold	Accept fair	Typical
	Empathy	Increased acceptance threshold	Accept unfair	Generous
Zhang et al. (2019)	Empathy	Avoid deviation from norm	Accept fair	Typical
Park et al. (2021)	Gratitude	Equitability for everyone	Accept unfair	Generous
Brandstätter and Königstein (2001)	Emotional stability	Reciprocity	Reject unfair	Typical
	Introversion	Reciprocity	Reject unfair	Typical
Weiß et al. (2020)	Extraversion	Increased acceptance	Accept unfair	Generous
Li and Chen (2012)	Agreeableness	Forgiveness	Accept unfair	Generous
Calvillo and Burgeno (2015)	Intuitive thinking	Heuristic thinking	Reject fair	Miserly
	Intuitive thinking	Heuristic thinking	Reject unfair	Typical
	Reflective thinking	Analytical thinking	Accept fair	Typical
	Reflective thinking	Analytical thinking	Accept unfair	Generous
Wei et al. (2022)	Intuitive thinking	System 1 and System 2 aligned	Accept fair	Typical
	Intuitive thinking	System 1 versus System 2	Reject unfair	Typical
Kawamura and Ogawa (2019)	Cognitive ability	Analytical thinking	Accept unfair	Generous
Stanley and Tran, 1998	Social norms	Social norm adherence	Accept fair	Typical
	Social norms	Social norm adherence	Reject unfair	Typical
Forgas and Tan (2013)	Negative mood	Self-interest tendencies	Reject unfair	Typical
	Positive mood	Altruism	Accept unfair	Generous
Wu and Gao (2018)	Proposer selfishness	Punish perceived selfishness	Reject fair	Miserly
Seier (2020)	Cognitive reflection	Intuitive thinking vs. reflective thinking	Accept fair	Typical
	Cognitive reflection	Intuitive thinking vs. reflective thinking	Reject unfair	Typical

#### 3.1.1 Emotions and affective states

Harmon-Jones et al. (2012) investigated how affect influences cognitive scope, particularly focusing on cognitive narrowing, wherein individuals tend to concentrate on specific details while not attending to broader information. Their study revealed that positive affect induces narrower categorizations, suggesting that individuals experiencing high-approach positive affect tend to adopt a more focused cognitive processing style, emphasizing specific aspects like fairness while giving lesser importance to broader considerations, such as potential gains. Conversely, negative affect prompted a more dispersed and broadly focused cognitive processing style. In the context of the Ultimatum Game, current literature (Riepl et al., 2016) emphasizes the significant impact of affective states on responder behavior. Specifically, participants experiencing positive affect were more inclined to accept unfair offers, while negative affect correlated with decreased acceptance rates of such offers.

##### 3.1.1.1 Negative affective states

Negative affective states, such as fear and guilt, play a significant role in shaping decision-making dynamics within the Ultimatum Game (Nelissen et al., 2011). Fear arises from the implicit desire to minimize personal risk, with apprehensions that low offers, considered unfair, may result in rejection. This led responders to adopt a more risk-averse approach by rejecting unfair offers more often, especially when there is a fear of negative evaluation if such an offer is accepted (Takagishi et al., 2016). Spite, characterized by harming others without immediate personal gain, influenced UG responders, who rejected perceived unfair offers out of spite despite lacking tangible benefits (Espín et al., 2015). Impatience, linked to a preference for immediate gratification over delayed rewards, drove some responders to reject low UG offers, thereby prioritizing instant gratification (Espín et al., 2015).

Additionally, anger plays a significant role in the UG. Responders may experience anger if they perceive offers as unjust or falling short of expectations, thereby influencing decision-making. However, the impact of anger may vary depending on play type, as participants may simulate their feelings and adjust behavior accordingly, potentially leading to constructive outcomes in rejections through specific strategic methods (Aina et al., 2020). Gilam et al. (2019) had similar findings across their Ultimatum Game paradigms, where angry responders rejected unfair offers.

##### 3.1.1.2 Positive affective states

Research using simulations of Ultimatum Game highlights the emergence of empathy in promoting cooperation in the Ultimatum Game (Iranzo et al., 2012). Higher levels of empathy within the group were associated with more equitable outcomes, thus a propensity for fairness for both the proposer and the responder. Emergence of empathy also implied an increase in the acceptance threshold of the responders, indicating acceptance of even the lower offers (Iranzo et al., 2012). Zhang et al. (2019) explored the evolution of fairness through empathy. They demonstrated that empathy might narrow the definition of equitable distribution and lead to responders being less tolerant of offers that deviate from this expectation.

A study on college students indicated that gratitude promotes cooperation and is an indicator of prosocial behavior, which led to more equitable outcomes for both the proposer and the responder (Park et al., 2021). The study found that participants were inclined to accept lower (or more unfair) offers as responders. The study suggested that this might be due to activation of brain regions associated with reward pathways and social cognition.

#### 3.1.2 Personality traits

Personality traits, reflecting individuals’ cognitive, emotional, and behavioral patterns, exhibit a notable degree of consistency and stability across time (Diener et al., 2019). In the context of decision-making processes within the Ultimatum Game, research has highlighted the influence of certain traits, like emotional stability, extraversion, and agreeableness, on game outcomes (Li and Chen, 2012; Brandstätter and Königstein, 2001).

Personality traits, such as emotional stability and extraversion, influenced responder behavior in the Ultimatum Game. Individuals exhibiting reciprocity—i.e., emotionally unstable and extroverted, or emotionally stable and introverted tendencies—were more inclined to reject unfair offers as a form of retaliatory response (Brandstätter and Königstein, 2001). In contrast, agreeableness significantly predicted forgiving behavior in economic games, highlighting the cognitive nature of forgiveness decisions beyond emotional influences (Li and Chen, 2012).

Weiß et al. (2020) demonstrated that individuals’ extraversion and neuroticism scores affected their UG decisions when facing a proposer with a dominant facial expression of anger. Highly extroverted individuals were more prone to accept unfair offers, whereas highly neurotic individuals were less inclined to do so. Despite some divergence in the literature, the observed impact of personality traits on UG outcomes underscores their role in shaping individuals’ cooperative tendencies within the game.

#### 3.1.3 Cognitive capabilities

Cognitive capabilities encompass mental attributes such as reasoning, problem-solving, and planning, which can significantly influence participants’ behavior in the Ultimatum Game (Gottfredson, 1997; Wei et al., 2022). Research has revealed the operation of two cognitive systems—intuitive and deliberative—in UG decision-making processes, with proposers primarily employing the deliberative system and responders utilizing a combination of both systems (Wei et al., 2022). Wei et al. (2022) have found that responders employ System 1 when rejecting an unfair offer, and employ both systems when accepting a fair offer. Seier (2020) found that responders engaging in more intuitive processes tend to exhibit prosocial behavior and punish selfish proposers.

Cognitive reflection (CR), the ability to step back from intuitive responses and engage in reflective thinking, plays a crucial role in UG behavior, with higher CR scores correlating with more selfish decisions (Czerwonka et al., 2018). Intuitive thinking was found to be linked to the rejection of UG offers, whereas reflective thinking was associated with accepting offers (Calvillo and Burgeno, 2015). Additionally, higher cognitive ability was found to be conducive to responders accepting lower (unfair) offers in the UG, underscoring the significant impact of cognitive ability on economic decision-making (Kawamura and Ogawa, 2019).

#### 3.1.4 Altruism and self-interest

Stanley and Tran (1998) conducted a study within a small liberal arts college environment, where students adhered closely to the social norm of fairness within the Ultimatum Game. The findings revealed that students overwhelmingly accepted offers deemed fair—those equal to or better than a 50/50 split—and consistently rejected offers considered unfair. This study elucidated the intricate interplay between self-interest and fairness, highlighting how individuals’ desire to be perceived as fair led to predictable behavior of accepting fair offers and rejecting unfair ones. Moreover, Forgas and Tan (2013) established a correlation between individuals’ moods and their self-interest tendencies. Negative emotions were associated with rejection of unfair offers and increased selfishness, while positive emotions were correlated with greater altruism, leading to acceptance of more unfair offers.

Altruism, defined as unselfishly helping others even at personal cost, emerged as a significant factor influencing responder behavior in the UG paradigm. In the exploration of altruism’s impact on children, Wu and Gao (2018) found that children were more inclined to punish in-group members for selfish behavior, challenging the theory of rational self-interest and highlighting their altruistic tendencies.

### 3.2 External factors

Table 2 summarizes the impact external factors have on the responder behavior in the Ultimatum Game.

**TABLE 2:** External factors affecting responder behavior.

Author	Factor name	Behavioral drive	Response to offer	Type of behavior
Dannenberg et al. (2012)	Ownership	Earned income	Reject unfair	Typical
Henrich et al. (2001)	Societal norms	Valuing fairness highly	Reject unfair	Typical
Capraro (2018)	Social preferences	Inequity aversion	Accept fair	Typical
	Social preferences	Inequity aversion	Reject unfair	Typical
Vavra et al. (2018)	Social norms	Valuing pre-existing norm	Accept unfair	Generous
Ciampaglia et al. (2014)	Power dynamic	Position perceived as weakness	Accept unfair	Generous
Yamagishi et al. (2009)	Power dynamic	Feeling of powerlessness, leading to retaliation	Reject unfair	Typical
Bolton and Zwick (1995)	Power dynamic	Lack of opportunity to punish	Accept unfair	Generous
Rodriguez-Lara (2016)	Power dynamic	No veto cost	Reject unfair	Typical
Roth et al. (1991)	Power dynamic	Favorable option elsewhere	Reject unfair	Typical
Sarlo et al. (2013)	Gender	Men in loss frame	Accept unfair	Generous
Li et al. (2018)	Attraction	Female responders	Accept unfair	Generous
Ma et al. (2017)	Attractiveness	Influence of proposer appeal	Accept unfair	Generous
Ma and Hu (2015)	Attractiveness	Influence of proposer appeal	Accept unfair	Generous

#### 3.2.1 Ownership

Early work demonstrated that anonymity and property rights substantially alter bargaining behavior in ultimatum game settings (Hoffman et al., 1994). Responders in the UG may be more sensitive to offers when they feel ownership over the initial sum. Dannenberg et al. (2012) found that responders who made the effort to earn the initial endowment led to greater rejection of an unequal offer as compared to a “no effort” condition where responders were assigned the initial endowment. This suggests that the perception of how the money was acquired can influence the responder’s sense of entitlement and their threshold for fairness in the division.

#### 3.2.2 Social norms

The empirical body of literature suggests that when people are aware of the societal norms that define what is fair, they are more likely to reject unjust offers. According to one study, people in societies that value fairness highly, like Japan, were more inclined to reject unjust offers (Henrich et al., 2001).

One paper explored the impact of social norms and found that fair offers were accepted and low offers were rejected because of inequity aversion (Capraro, 2018). Similarly, a meta-analysis on the Ultimatum Game showed that social preferences had a major impact on responder behavior because responders were more likely to reject unfair offers when they anticipated being watched by others (Oosterbeek et al., 2004).

Recent findings demonstrate that the average of expected offer determined the threshold of acceptability, while the expected variance of the offer distribution influenced the strictness of this threshold. Participants were more inclined to accept lower offers when the expected variance of offer was high. According to this study, social expectations are more complex than what existing theories predict (Vavra et al., 2018).

#### 3.2.3 Power dynamics

The responder typically has lesser power than the proposer in the UG. This power dynamic can significantly influence how the responder behaves and makes decisions. Literature indicates that when responders perceived their position as “weak” or “weaker,” they were more likely to accept unfair offers (Ciampaglia et al., 2014). In contrast, the study also showed that when the responder felt powerless and they had to accept begrudgingly, they engaged in retaliatory behavior. These results are concordant with previous literature (Yamagishi et al., 2009).

Yamagishi et al. (2009) showed that responders deliberately rejected unfair offers. This is not because of the prosocial behavior mentioned in the previous section. Instead, it was an attempt to avoid imposing an inferior status. Rodriguez-Lara (2016) also posited that responders are more likely to reject offers when the rejection cost is not that high and that responders demand more when they believe they have more power.

Numerous studies have investigated variations of the Ultimatum Game, namely the No-Veto Cost Game and the Impunity Game, which constitute a rule-based way of altering the power dynamics of the experimental framework. The general trend of the results showed that responders were most likely to reject unfair offers in the Ultimatum Game and least likely to reject unfair offers in the Impunity Game (Bolton and Zwick, 1995). The acceptance rate for fair offers in the Ultimatum Game remained the highest (Camerer and Thaler, 1995), while the No-Veto Cost Game showed relatively lower rates. This suggests that while fairness aligns with expectations, the potential for future unfairness and the cost of punishment might influence some responders (Fehr and Gächter, 2002).

Outside options ensure that the responder has other opportunities if a negotiation is not successful. They play a role in influencing responder behavior by providing them a “safety net,” thus ensuring greater power for the responder. Empirical research suggests that people are more inclined to reject unjust offers when they have other, more favorable options. Roth et al. (1991) found that when participants knew they might play the game with someone else who might be more likely to make a fair offer they were more inclined to reject unfair offers.

#### 3.2.4 Gender and attraction

Numerous studies have investigated the influence of gender and attraction on ultimatum bargaining. According to this research, men and women generally choose differently. Men were more likely to reject low offers and women were more likely to accept them (Li et al., 2018). Sarlo et al. (2013) observed that in a loss frame men accept more unfair offers than women. Various reasons such as differing degrees of risk aversion, varying degrees of empathy and varying social norms have been suggested as causes for these decision-making disparities (McGee and Constantinides, 2013; Sarlo et al., 2013).

Attraction has also been found to have an impact on decision-making in the UG. According to a set of studies (Ma et al., 2017; Ma and Hu, 2015), people are more inclined to accept cheap proposals from attractive than from unattractive proposers. This suggests that people’s perceptions of an offer’s fairness may be influenced by the proposer’s appeal.

The findings of these studies suggest that gender and attraction can both play a role in decision-making in the Ultimatum Game. These findings have important implications for understanding how people interact with each other in social and economic situations.

## 4 Discussion

An examination of factors influencing responder behavior reveals insights into how internal and external variables modulate bargaining behavior. Previous empirical studies have established consistent patterns in player responses to specific factors. However, integrating these factors unveils important information about responder behavior patterns and the underlying neural substrates. Developing a framework to distinguish between typical and atypical behaviors, and understanding the factors that facilitate each would advance our comprehension of bargaining behavior within the Ultimatum Game microcosm.

Recent computational research suggests that behavior in the Ultimatum Game is not only driven by fixed traits or stable preferences. Instead, internal influences appear as shifting cognitive and emotional processes that evolve throughout the interaction. Heffner et al. (2021) show that responders track emotion prediction errors, meaning the gap between expected and experienced feelings. These discrepancies lead to changes in acceptance or rejection patterns that cannot be fully accounted for by reward-focused models. Kim et al. (2024) argue that the way emotions unfold during bargaining can classify responders with surprising accuracy. Distinct emotional pathways are linked to generosity, reciprocity, or punitive rejection.

Viewed together, these findings extend the usual internal and external factor framework. They indicate that responders are continuously updating their feelings, interpreting social cues, and adjusting their decisions in real time. Typical and atypical behavior may therefore reflect differences in emotional learning and the course that emotions take throughout the exchange, rather than personality alone. Overall, this work points to responder behavior as a dynamic product of ongoing affective computation, social expectations, and interpretations of the proposer’s intent.

Distinguishing between rational and typical players is essential. A rational player accepts any offer, recognizing the value of receiving something over nothing (Camerer, 2003). In contrast, “typical responders,” as proposed in this paper, are players who accept fair offers and reject unfair ones. They view fair offers as equitable, aligning with their internal fairness standards (Güth et al., 1982), while they reject unfair offers due to a sense of injustice (Camerer, 2003).

In the Ultimatum Game, decision-making involves factors beyond fairness, including internal and external influences. Previous research has revealed participants exhibiting atypical behavior–accepting unfair offers and rejecting fair ones–lead to the classification of “atypical responders.” While typical players consistently accept fair offers and reject unfair ones, atypical players deviate from this pattern, including “miserly responders” who reject fair offers and “generous responders” who accept unfair ones. This classification enhances our understanding of diverse behavioral responses, as detailed in Table 3.

**TABLE 3:** Types of responders in the ultimatum game.

Response/type of offer	Fair offer	Unfair offer
Accept	Typical responder	Generous responder
Reject	Miserly responder	Typical responder

**FIGURE 1:**
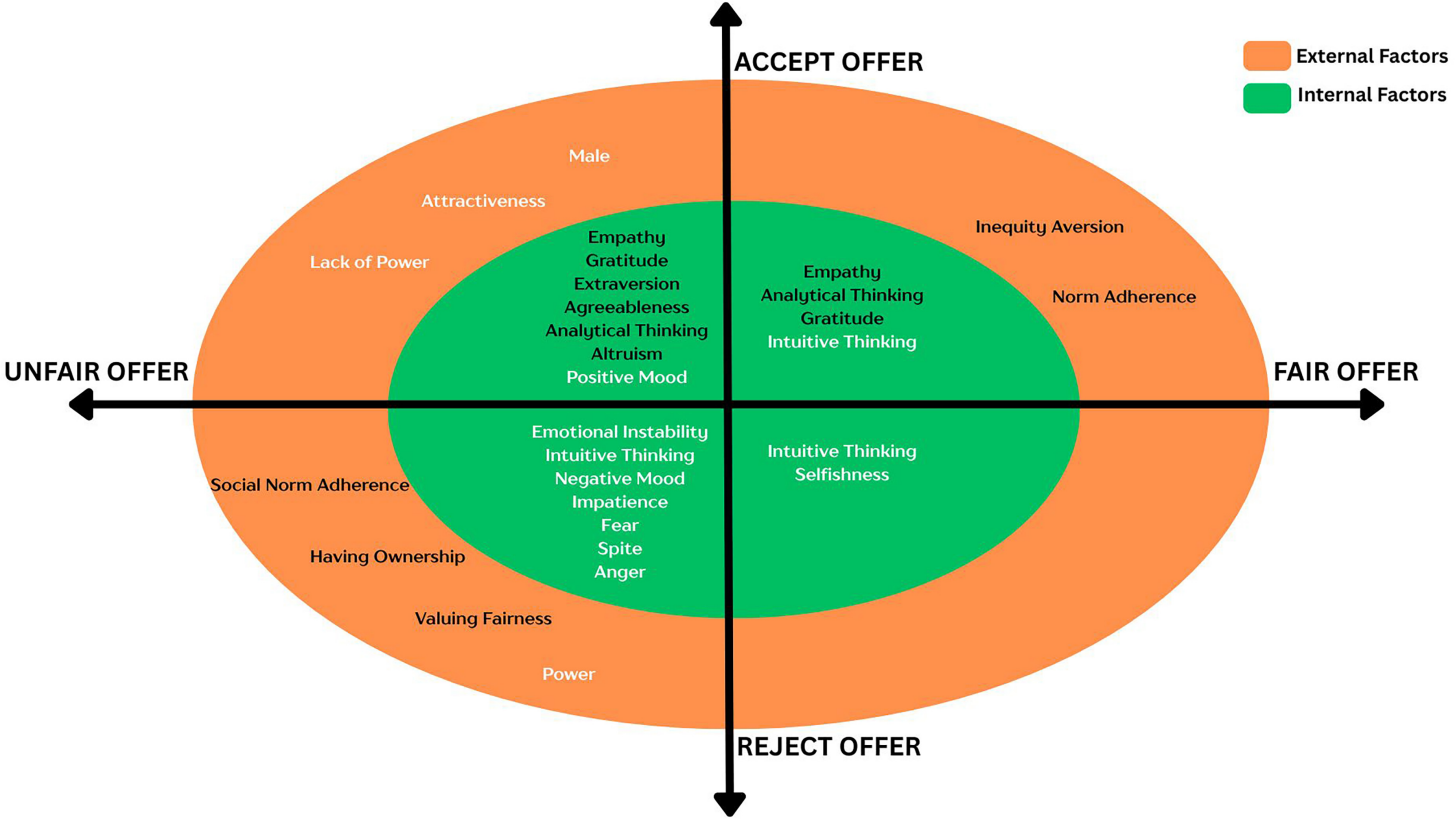
Conceptual map of factors affecting responder behavior.

This characterization of players can be further elaborated on by creating a matrix of how various factors are instrumental in creating these players. An analysis of the preceding section’s findings affords insight into how distinct factors contribute to the inclination of a player toward a particular grouping. Understanding what causes typical gameplay is important because it helps one grasp the dynamics of transactions, negotiations, and agreements in the game. As discussed earlier, the synthesis of diverse factors is systematically organized and presented in Figure 1, delineating their respective categories and the behavioral manifestations they potentiate.

In the four-quadrant figure, the *X*-axis delineates the nature of the offer (fair or unfair), while the *Y*-axis represents the responder’s reaction to the said offer (acceptance or rejection). Each quadrant is characterized by juxtaposing two concentric circles, designating internal (intrinsic) and external (extrinsic) factors, thereby providing a nuanced understanding of responder behavior.

Quadrant one, representing the behavior of accepting fair offers, exemplifies typical responders. These responders are driven by both analytical thinking and intuitive thinking, as well as positive emotions like empathy and gratitude. The external factors that drive this behavior are inequity aversion and norm adherence. Quadrant two represents the behavior of accepting unfair offers, that shapes “Generous Responders,” and this behavior is driven internally by empathy, gratitude, extraversion, agreeableness, altruism, analytical thinking, positive mood and external factors like gender, attractiveness, and lack of power. Quadrant three depicts behavior of typical responders who reject unfair offers, and these behaviors may be driven by negative emotions like fear, spite, impatience, anger, emotional instability, negative mood and intuitive thinking. The external factors include ownership, adherence to social norms, valuing fairness and having power. The least explored is quadrant four, where behaviors can be grouped as “Miserly Responders,” and here the behaviors are driven by intuitive thinking and selfishness.

The human frontal pole is responsible for higher-order cognition, complex decision-making, social behavior and goal management (Bludau et al., 2014; Orr et al., 2015; Rilling and Sanfey, 2011). The medial frontal pole underlies affective processing and social cognition (Orr et al., 2015), while the lateral frontal region modulates cognition and reasoning (Chau et al., 2025). Kahneman’s dual system theory distinguishes between the quick, intuitive System 1 and slow, deliberative System 2. System 1 may supposedly be linked to the functioning of the medial frontal region as instinctual behavior and emotional functions are closely associated with System 1 (de la Vega et al., 2016). In contrast, the more deliberative System 2 may be closely linked to the functioning of the lateral frontal pole (Hartwigsen et al., 2021). This perspective attributes factors, such as fear, impatience and selfishness to the functioning of System 1 as they are crucial for survival reasons. These functions are more emotional and intuitive, and known to be associated with medial frontal regions, such as amygdala, hippocampus, hypothalamus and basal ganglia (Kerahrodi and Michal, 2020; Šimić et al., 2021). Higher order factors, such as empathy and altruism, need slow deliberation and may be attributed to the functioning of System 2. These functions are associated with the lateral frontal regions. The lateral frontal region forms an important segment of the lateral fronto-parietal network that is responsible for supporting complex cognitive operations (Hartogsveld et al., 2018; Vendetti and Bunge, 2014).

In the figure, it is instructive to note that all factors inscribed in white relate to the functioning of System 1 and those in black refer to System 2. An initial examination of the diagram reveals the relative lack of factors that characterize decision-making for a fair offer. This may be because the fairness perspective is relatively more standard and unambiguous. However, the decision made for an unfair offer—either acceptance or rejection—is unmistakably divided across a wide range of internal and external factors.

While exploring and characterizing neural substrates of the internal factors, first all factors leading to rejection (fair or unfair offer) can be aligned with System 1 which comprises some of the “older” parts of the brain (Kahneman, 2011), while all factors leading to acceptances (fair or unfair offer) can be broadly aligned with System 2’s working. Acceptance of a fair offer is the only instance where an interaction of System 1 (intuitive thinking) and System 2 (analytical thinking) is involved. Bear and Rand (2016) explain this anomaly as possible due to the convergence of the fairness norm with self-interest. The characterization of external factors to brain regions is also distinct. Factors leading to typical behavior (accept fair and reject unfair offer) are initially mediated by System 2. Only power, leading to the rejection of unfair offers, can be attributed to the working of System 1. Also, factors responsible for atypical behavior (acceptance of unfair offer) are mediated by the System 1.

Wei and others demonstrated that the deliberative system typically processes and accepts unfair offers. This assertion is further bolstered by previous research suggesting that the intuitive, automatic System 1 is predominantly responsible for rejecting unfair offers, often motivated by emotions such as anger or spite (Aina et al., 2020; Espín et al., 2015). To elucidate the underlying rationale, it is imperative to contextualize bargaining within an evolutionary framework. Researchers argue that human cooperation likely evolved as an adaptive strategy for survival and reproductive success (Henrich and Muthukrishna, 2021).

Prehistoric bargaining presumably diverged significantly from its contemporary counterpart, characterized by a lesser reliance on verbal communication and a greater emphasis on reciprocity. Transactions involving tools, food, or mates may have constituted implicit agreements rooted in past interactions (Richerson et al., 2003). Trust and reputation, cultivated through repeated engagements, likely held paramount importance (Trivers, 1971). Given the probable absence of intricate linguistic abilities, prehistoric bargaining likely heavily leaned on the intuitive systems of the old brain for several reasons. With complex language not as prevalent, swift decision-making would have been imperative. System 1’s superior processing speed facilitated rapid assessments, particularly critical in potentially precarious scenarios such as prehistoric exchanges (Bechara et al., 1994). Moreover, non-verbal communication likely assumed a pivotal role. System 1’s adeptness in deciphering emotions conveyed through facial expressions and body language would have been instrumental in evaluating trustworthiness and assessing potential threats during negotiations (Adolphs et al., 1994).

Within the context of the Ultimatum Game, System 1 operates to either retaliate against the proposer, as evidenced by manifestations of fear, spite, and anger in quadrant three, or evidenced as a reaction to power and attractiveness of the proposer. Retaining its inherent attributes of rapid processing, emotional acuity, and social instincts, System 1 serves as an innate compass guiding human decision-making processes. However, with the passage of time, the growing complexities of social dynamics and capitalist structures presented novel challenges to the processing capabilities of the old brain (Eyre et al., 2024). The proliferation of data, intricate contractual arrangements, and abstract financial mechanisms strain the old brain’s proclivity for swift decision-making. Moreover, System 1’s inclination toward immediate gratification and present-focused rewards may prove disadvantageous in contexts requiring long-term planning and comprehension of abstract concepts such as derivatives (Camerer et al., 2004). Consequently, evolution has recognized the necessity for a more sophisticated deliberative system led by the prefrontal cortex—the new brain or System 2—which transcends mere survival imperatives. This system is geared toward facilitating higher-order cognitive functions that set humans apart from other species (Mattson, 2014), including executive functioning, self-regulation, and social cognition (Diamond, 2013).

Thus, decision-making processes inherent in the Ultimatum Game heavily rely on these cognitive functions. In the context of accepting unfair offers (quadrant two), the System 2 is instrumental in executing higher-order functions that counteract the initial inclination of System 1 to reject unfair offers, thereby superseding or altering the initial response of the old brain. Factors such as altruism, empathy and gratitude have the capacity to override the initial reactions of System 1 to the proposed offer. Individuals exhibiting high levels of agreeableness or those experiencing positive mood may be more inclined to accept an unfair offer to mitigate potential social conflict (Park et al., 2021; Li and Chen, 2012). Additionally, individuals with extraverted personalities characterized by heightened reward sensitivity may be swayed by the immediate gratification associated with receiving any portion of the monetary offer, thereby disregarding its unfairness (Brandstätter and Königstein, 2001). Cognitive abilities also emerge as significant factors in overriding the initial response to fairness based on underlying self-interests (Kawamura and Ogawa, 2019). In terms of externally-driven factors, attractiveness of the proposer and lack of power supersede the drive to reject unfair offers due to a reduced salience of the fairness perspective (Ma et al., 2017; Yamagishi et al., 2009). Lastly, males were observed to accept unfair offers more than females in a loss frame due to elicitation of a greater defensive response (System 1 elicitation) in males as measured by increase in skin conductance and heart rate (Sarlo et al., 2013).

Quadrant three is implicated by both Systems 1 and 2. Emotions such as fear, anger and spite internally drive the rejection of an unfair offer. The influence of power is also very evident in driving the intuitive decision to reject, in a way to assert dominance over the proposer. However, external factors such as social norm adherence, valuing of fairness and ownership, driven by System 2, are also responsible for said decision. It is important to note that playing reject to an unfair offer may not necessarily be an intuitive decision, it also has strategic importance for long-term survival in the game (Bahry and Wilson, 2006). Hence, the presence of externally-driven decisions indicate that deliberative thinking is equally involved in the decision to reject an unequal offer.

From an economic standpoint, it is logically sound for a player to accept any offer above zero. Mussel et al. (2013) contend that according to rational decision theory, individuals with a higher propensity for cognitive engagement are more inclined to accept a greater number of offers. However, this portrayal only captures one facet of the phenomenon. While the act of accepting unfair offers is predominantly associated with System 2 and rejecting fair offers with System 1, the interplay between these systems is considerably more intricate than previously perceived. A comprehensive review on the evolution of fairness within the UG underscores that while traditional economic models predicate behavior on rational self-interest, empirical findings indicate a pronounced inclination toward fairness (Rand et al., 2013).

A wealth of research asserts the evolutionary advantages of utilizing System 2. Our expanded brain size, characterized by an augmented number of cortical neurons, facilitated intricate social interactions and tool utilization, confer an evolutionary edge (Herculano-Houzel, 2016). Humans understand fairness norms and can anticipate others’ intentions by relying on mentalizing capacities and social-cognitive networks in the brain (Frith and Frith, 2006). This cognitive progression enabled humans to adeptly navigate diverse environments and flourish as a species (Pittella, 2024), laying the groundwork for economic innovation and resource management, further bolstering human prosperity (Eyre et al., 2024). Based on Kahneman’s dual system theory and the empirical work done on cognitive reflection (Frederick, 2005), various perspectives offer insights into the evolutionary roots and modern cognitive processes guiding decision-making. However, is it invariably advantageous to employ the new brain? Consider a scenario where you, as the responder, encounter proposers singularly focused on self-interest. Accepting offers based on self-interest could potentially expose you to exploitation, with proposers consistently offering diminished amounts in subsequent rounds, thereby yielding diminished overall gains. Conversely, reliance solely on the old brain in such situations proves equally disadvantageous. Rejecting offers based on the fairness norm could lead to a sub-optimal strategy over time. Hence, while evolution underscores the indispensability of the new brain, it advocates for a balanced interplay between the two to avoid both exploitation and selection of sub-optimal strategies.

## 5 Implications

The delineation between System 1 and System 2 in decision-making processes, particularly within the framework of the Ultimatum Game, carries significant implications for understanding human behavior and societal dynamics. By recognizing the distinct roles played by evolutionary neural substrates, researchers gain insight into the underlying mechanisms that govern responses to fairness, reciprocity, and self-interest. Neuroimaging evidence suggests that the brain computes social and moral value using domain-general valuation systems, integrating fairness-related considerations into choice behavior (Tusche et al., 2016). This understanding could inform various fields, including economics, psychology, and evolutionary biology, by providing a nuanced perspective on how ancient survival instincts interact with modern cognitive processes in shaping social interactions and economic transactions.

Traditional economic models often assume purely rational actors. However, the framework suggests emotional responses from System 1 (e.g., anger at unfair offers) can influence economic decisions (Aina et al., 2020). This challenges the rationality assumption and necessitates incorporating emotional factors into economic models (Camerer, 2003). The framework also sheds light on the neural correlates of fairness and cooperation. System 1’s role in emotional responses to fairness violations aligns with research on the amygdala’s involvement in processing unfairness (Stallen et al., 2018). Additionally, System 2’s involvement, as linked with executive control and goal maintenance (Miller and Cohen, 2001), may explain why some individuals prioritize fairness even when accepting unfair offers (Park et al., 2021). Meta-analytic evidence from neuroimaging studies of the Ultimatum Game confirms consistent involvement of affective and control-related brain systems during responses to unfair offers (Gabay et al., 2014). Lastly, the framework suggests an evolutionary basis for decision-making. The old brain’s emphasis on rapid reactions might have been crucial for survival in simpler environments (Bechara et al., 1994). Conversely, the new brain’s higher-order functions likely emerged later, allowing for more complex social interactions and economic strategies (Herculano-Houzel, 2016).

## 6 Limitations

Despite its insights, the model presented in this paper has its limitations. Firstly, the functional dichotomy between the brain structures associated with medial versus lateral frontal poles oversimplifies the complexity of neural processes underlying decision-making. Ordinarily, brain regions do not perfectly map on to either of the systems and the same region can serve different functions based on task demands (Hiser and Koenigs, 2018; McDonald et al., 2020). The System 1/System 2 categorization is a useful heuristic for conceptual purposes rather than an accurate mapping. Additionally, the evolutionary perspective presented here may overlook the influence of cultural, social, and environmental factors on decision-making behaviors. Future research should aim to integrate these factors into the model to provide a more comprehensive understanding of human decision-making.

## 7 Conclusion

The exploration of decision-making processes through the System 1/System 2 framework illuminates fundamental aspects of human behavior and cognition. By delineating between ancient survival instincts and modern cognitive functions, this model provides valuable insights into how individuals navigate social interactions and economic transactions. While System 1 governs initial responses rooted in primal emotions and instincts, System 2 enables higher-order cognitive functions necessary for complex decision-making in modern society. This dichotomy underscores the evolutionary continuity in human behavior while highlighting the adaptive nature of cognitive processes. However, this model’s simplicity may overlook the influence of cultural, social, and environmental factors on decision-making. Future research should endeavor to integrate these complexities for a more comprehensive understanding. Nevertheless, this framework offers a valuable lens through which to analyze human behavior across disciplines, with implications for economics, psychology, and evolutionary biology. By refining this model and incorporating diverse perspectives, we can deepen our understanding of decision-making processes and their implications for individuals and societies in an increasingly complex world.

## Data Availability

The original contributions presented in this study are included in this article/supplementary material, further inquiries can be directed to the corresponding author.
